# CD36 regulates substrates utilisation in brown adipose tissue of spontaneously hypertensive rats: In vitro study

**DOI:** 10.1371/journal.pone.0283276

**Published:** 2023-04-13

**Authors:** Jan Silhavy, Petr Mlejnek, Miroslava Šimáková, Irena Marková, Hana Malínská, Martina Hüttl, Ludmila Kazdová, Dmitry Kazantsev, Massimiliano Mancini, Jiří Novotný, Michal Pravenec

**Affiliations:** 1 Institute of Physiology, Czech Academy of Sciences, Prague, Czech Republic; 2 Institute for Clinical and Experimental Medicine, Prague, Czech Republic; 3 Department of Physiology, Faculty of Science, Charles University, Prague, Czech Republic; 4 1st Faculty of Medicine, Institute of Pathological Physiology, Charles University, Prague, Czech Republic; 5 Division of Morphologic and Molecular Pathology, S. Andrea Hospital, Sapienza, University of Rome, Rome, Italy; The Chinese University of Hong Kong, HONG KONG

## Abstract

Thermogenesis in brown adipose tissue (BAT) uses intracellular triglycerides, circulating free fatty acids and glucose as the main substrates. The objective of the current study was to analyse the role of CD36 fatty acid translocase in regulation of glucose and fatty acid utilisation in BAT. BAT isolated from spontaneously hypertensive rat (SHR) with mutant *Cd36* gene and SHR-*Cd36* transgenic rats with wild type variant was incubated in media containing labeled glucose and palmitate to measure substrate incorporation and oxidation. SHR-*Cd36* versus SHR rats showed significantly increased glucose incorporation into intracellular lipids associated with reduced glycogen synthase kinase 3β (GSK-3β) protein expression and phosphorylation and increased oxidation of exogenous palmitate. It can be concluded that CD36 enhances glucose transport for lipogenesis in BAT by suppressing GSK-3β and promotes direct palmitate oxidation.

## Introduction

Brown adipose tissue (BAT) plays an important role in maintaining body temperature by producing heat through uncoupling oxidative phosphorylation from ATP production. BAT was detected also in adult humans and because of its important involvement in energy metabolism potential role of BAT in the pathogenesis of obesity and type 2 diabetes are the subject of intense interest [1–3]. Using a systems genetics analysis in BXH/HXB recombinant inbred (RI) strains, derived from SHR (spontaneously hypertensive rat) and BN (Brown Norway) progenitors, we identified a quantitative trait locus (QTL) associated with BAT relative weight on chromosome 4 [4]. This QTL overlapped with a coexpression module eigengene QTL containing candidate genes with mRNA abudance regulated in *cis* and correlated with BAT relative weight. The *Cd36* (fatty acid translocase) gene was a highly connected hub gene of the coexpression module associated with relative BAT weight [4]. The SHR harbors a deletion variant of *Cd36* gene [5] which predisposes this strain to insulin resistance, dyslipidemia and increased blood pressure [6–10]. Mutated *Cd36* thus represents a prominent candidate gene for QTL associated with BAT relative weight and function.

Thermogenesis in BAT uses intracellular triglycerides, circulating free fatty acids and glucose as the main substrates. Glucose that enters brown adipocytes is used mainly for lipogenesis and plays only a minor role in BAT thermogenesis compared to fatty acids [11]. It was reported that fatty acids synthesised from glucose, as well as fatty acids transported into brown adipocytes, are not directly used as fuel but instead are used to replenish intracellular triglyceride stores from which fatty acids are provided by lipolysis during thermogenesis [12, 13]. Uptake of exogenous fatty acids by BAT is mediated by several transporters including CD36 fatty acid translocase. In the current study, we tested the hypothesis that *Cd36* regulates fuel utilisation in BAT in the SHR. Results of the current study provided compelling evidence for an important role of *Cd36* in enhancing glucose transport and utilisation and direct oxidation of exogenous palmitate in BAT.

## Methods

### Animals

The SHR/OlaIpcv strain (referred to as SHR) and the SHR/Ola-TgN(EF1a*Cd36*)19Ipcv transgenic line TG19 (referred to as SHR-*Cd36*) [9] were housed in an air-conditioned animal facility at 23°C and 12 h light/12 h dark cycle and allowed free access to Sniff^®^ R-Z standard laboratory chow (ssniff Spezialdiäten GmbH, Soest, Germany) and water. These strains are genetically identical except for the expression of wild type *Cd36* transgene under control of universal EF-1α promoter in the transgenic line. Biochemical, metabolic and morphometric phenotypes in both strains were assessed in 3-month-old non-fasted male rats (N = 8 per strain). All experiments were performed in agreement with the Animal Protection Law of the Czech Republic and were approved by the Ethics Committee of the Institute of Physiology of Czech Academy of Sciences, Prague (protocol number 15-2022-P).

### Glucose oxidation and incorporation into BAT lipids

After decapitation in the non-fasted state, interscapular BAT was dissected and 60 mg were incubated for 2 hours in Krebs-Ringer bicarbonate buffer with 5 mmol/L glucose alone or together with 0.5 mmol/L palmitate, 0.1 μCi [U-^14^C] glucose/ml and 2% bovine serum albumin, gaseous phase 95% O_2_ and 5% CO_2_ in the presence of 250 μU/ml insulin in the incubation media. Glucose oxidation was determined in BAT by measuring the incorporation of [U-^14^C] glucose into CO_2_. For measurement of incorporation of radiolabeled glucose into lipids, at the end of incubation, BAT was removed from media, rinsed in saline, transferred into chloroform:methanol (2:1), lipids were extracted and radioactivity measured [14].

### Palmitate oxidation and incorporation into BAT lipids

Isolated BAT (60 mg) was incubated in Krebs-Ringer bicarbonate buffer with 0.5 mmol/ml palmitic acid alone or together with 5 mmol/L glucose, 0.5 μCi/mL of ^14^C-palmitic acid and 2% bovine serum albumin, gaseous phase 95% O_2_ and 5% CO_2_ in the presence of 250 μU/ml insulin in the incubation media. Palmitate oxidation was determined in BAT by measuring the incorporation of [U-^14^C] palmitate into CO_2_. For measurement of incorporation of radiolabeled pamitate into lipids, at the end of incubation, BAT was removed from media, rinsed in saline, transferred into chloroform:methanol (2:1), lipids were extracted and radioactivity measured [14].

### SDS-PAGE and Western blotting analysis

BAT was homogenised in RIPA buffer complemented with protease and phosphatase inhibitors (Sigma Aldrich). The tissue was lysed at 4°C for 30 min with gentle agitation and then centrifuged at 14000 x g for 15 min. The supernatant was collected while avoiding the layer of fat and used for Western blotting as described previously [15]. Samples from each group were run on the same gel. They were resolved on 10% polyacrylamide gels, electrotransferred to a nitrocellulose membrane, and after blocking with 5% skim milk, incubated overnight (at 4°C) with the following primary antibodies: GSK-3β, phospho-GSK-3β (Ser 9) and β-actin (Santa Cruz Biotechnology), IRβ, Akt and phospho-Akt (Ser 473) (Cell Signaling Technology) and phospho-IRβ (Tyr 1361) (Abcam). Membranes were then washed and incubated with the appropriate HRP-conjugated secondary antibody. Blots were exposed to X-ray film, scanned with a high-resolution CCD scanner (EPSON Perfection V600 Photo), and immunochemical signals were quantified by densitometric analysis using ImgeJ software and normalised to total protein determined by Ponceau S staining. At least three separate experiments were performed for each determination.

### Gene expression determined by real-time PCR

Total RNA was extracted from interscapular BAT using Trizol reagent (Invitrogen) and cDNA was prepared and analysed by real-time PCR testing using QuantiTect SYBR Green reagents (Qiagen, Inc.) on an Opticon continuous fluorescence detector (MJ Research). Gene expression levels were normalised relative to the expression of the peptidylprolyl isomerase A (*Ppia*) (cyclophilin) gene, which served as the internal control. The results were determined in triplicates. Primers used for the validation of differentially expressed genes selected from significant pathways are given in S1 Table.

### Histological analysis

Brown adipose tissue harvested from both SHR and SHR-*Cd36* rats (n = 6 for each group) was formalin fixed and paraffin embedded. Multiple sections were cut from each block and stained both with Hematoxilin & Eosin for quality assessment and with immunoperoxidase stain with mouse monoclonal [TLD-3A12] to CD31antibodies (Abcam plc, Cambridge UK) for capillary evaluation. Stained sections were exmined by an observer blindend by the groups and ten high power fields from each rat were aquired using a digitalized microscope camera. Images were analysed with ImageJ 1.8.0 (NIH, Bethesda) image analysis software using a color threshold method and automated capillary density measurement.

### Statistical analysis

The data are expressed as means ± SEM. Individual groups were compared by Student t-test. Normality of distribution was tested by Shapiro-Wilk method. Statistical significance was defined as P<0.05. Two-way ANOVA was used to test for *presence of substrates in media* x *Cd36 genotype* interactions. For variables showing evidence of interaction, the Holm-Sidak test which adjusts for multiple comparisons was used to determine whether the effects of substrates in media were significant in the SHR strain and in the SHR-*Cd36* transgenic strain. Significant difference in blood capillary number in BAT was evaluated by Student t test.

## Results

### *Cd36* regulates fuel utilisation in BAT

**Fig 1:**
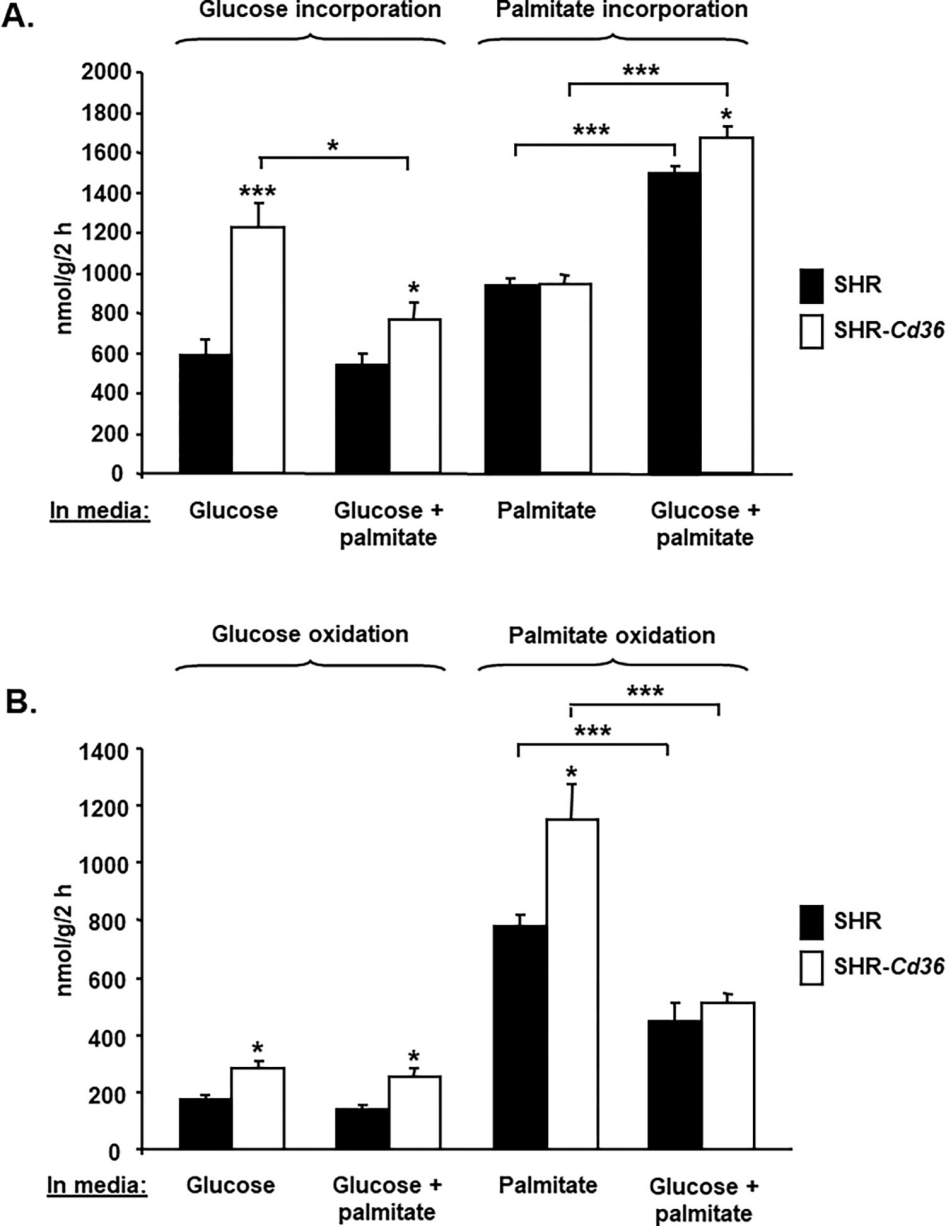
Effects of *Cd36* on substrate utilisation in BAT. A. Ex vivo glucose and palmitate incorporation into BAT lipids in SHR-*Cd36* transgenic versus SHR rats. B. Ex vivo glucose and palmitate oxidation in BAT from SHR-*Cd36* transgenic versus SHR rats. Two-way ANOVA results: P values of statistical significance for effects strain (*Cd36* genotype), type of incubation media (glucose/palmitate) and strain x substrate interaction. For pairwise multiple comparison procedures Holm Sidak testing was used. * and *** denote P<0.05 and P<0.001, respectively.

**Fig 2:**
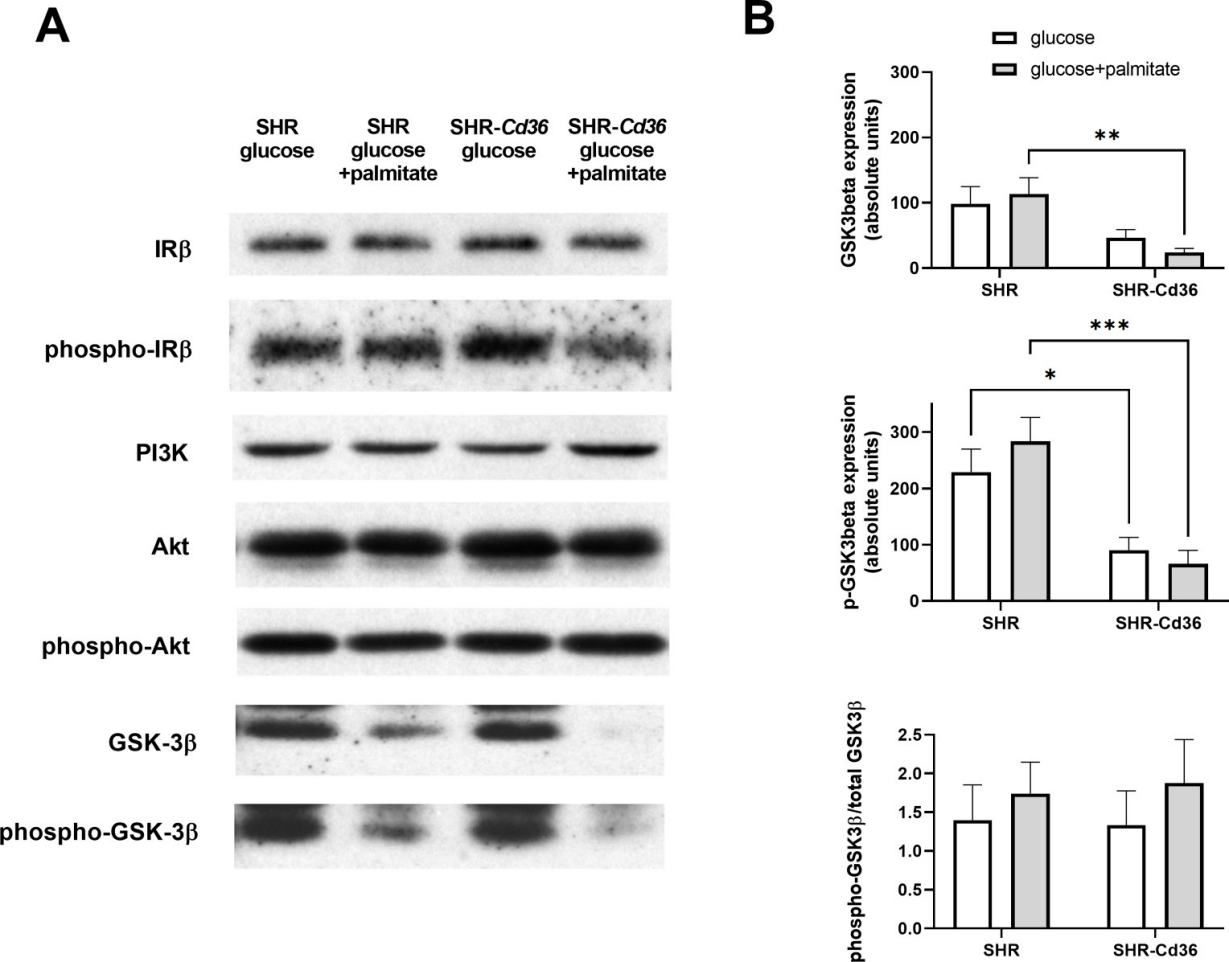
Effect of *Cd36* on expression and phosphorylation of key components of the insulin signalling pathway. Samples of BAT isolated from SHR and SHR-*Cd36* were incubated in Krebs-Ringer bicarbonate buffer with glucose alone or together with palmitate, then homogenised and subjected to gel electrophoresis and Western blotting as described in Methods. Immunoblots shown are representative of six experiments (A). Signal intensities corresponding to detected proteins were quantified by densitometric analysis and normalised to total protein determined by Ponceau staining (B). *, **, and *** denote P<0.05, P<0.01, and P<0.001 significant differences.

BAT isolated from SHR and SHR-*Cd36* rats was incubated in media containing glucose or palmitate or both substrates and incorporation of radioactively labeled glucose and palmitate into intracellular lipids and into CO_2_ was measured. In BAT from SHR-*Cd36* versus SHR rats that was incubated in media with glucose alone, glucose incorporation into intracellular lipids (lipogenesis) was significantly increased (Fig 1A) which was associated with reduced GSK-3β protein expression and phosphorylation (Fig 2). These findings suggest that *Cd36* enhances glucose transport and lipogenesis in BAT by suppressing GSK-3β. When palmitate was added to glucose in incubation media, incorporation of glucose into BAT lipids was reduced, most likely due to the fact that triglycerides were synthesised from added palmitate and relatively less glucose was needed for lipogenesis. In addition, added palmitate had no effects on the expression and phosphorylation of GSK-3β (Fig 2). Palmitate incorporation into intracellular lipids in BAT from SHR-*Cd36* versus SHR rats was significantly increased only when BAT was incubated in media containing both palmitate and glucose while no difference was observed when BAT was incubated in media with palmitate alone (Fig 1A). Glycerol-3-phosphate necessary for synthesis of intracellular triglycerides in BAT incubated in media with palmitate without glucose must be provided by glyceroneogenesis. When BAT is incubated in media containing both glucose and palmitate, glycerol is provided by both glyceroneogenesis and glycolysis and thus incorporation of palmitate into BAT lipids was increased (Fig 1A). These findings suggest that *Cd36* is needed for glycerol production for triglyceride synthesis by glycolysis (most likely by enhancing glucose transport) but not for glyceroneogenesis.

Glucose oxidation in BAT from SHR-*Cd36* versus SHR rats was significantly increased independent on presence of palmitate in media but overall smaller when compared to palmitate oxidation (Fig 1B). This observation suggests that glucose in BAT is preferentially used for lipogenesis, not for oxidation. On the other hand, BAT from SHR-*Cd36* versus SHR rats showed significantly increased palmitate oxidation when incubated in media with palmitate alone (Fig 1B). The fact that BAT from SHR-*Cd36* rats incubated in media with palmitate alone showed similar palmitate incorporation into intracellular lipids (Fig 1A) but significantly increased palmitate oxidation (Fig 1B) provides evidence that in the presence of functional CD36 exogenous palmitate is directly oxidised in BAT rather than incorporated into intracellular triglycerides. When BAT was incubated in media containing both glucose and palmitate, oxidation of palmitate was not different between SHR-*Cd36* and SHR. This result suggests that in the presence of glucose, palmitate is preferentially incorporated into triglycerides rather than oxidised.

### Effects of substrates in incubation media on expression of genes and proteins involved in glucose metabolism and insulin signalling in BAT

Since SHR-*Cd36* versus SHR rats showed significantly increased glucose incorporation into BAT lipids (lipogenesis) in the presence of insulin, we tested whether *Cd36* affects insulin signalling by analysing expression and phosphorylation of selected proteins from phosphatidylinositol 3-kinase-Akt, the main signalling pathway downstream of insulin. As can be seen in Fig 2, protein expression and phosphorylation of IRβ (insulin receptor β), PI3K (phosphoinositide 3-kinase), and AKT (protein kinase B, PKB) proteins showed no significant differences between the SHR-*Cd36* versus SHR strains and substrates in media. On the other hand, the expression of GSK-3β (glycogen synthase kinase 3β) was significantly reduced in the presence of wild type *Cd36* though independently on substrates in incubation media. The amount of phosphorylated GSK-3β was reduced to similar extent and the ratio of phosho-GSK-3β/GSK-3β was not changed. Images of full-length immunoblots and Ponceau staining of total proteins bound to nitocelulose membranes used for quatification are provided in the (S1-S7 Figs in S1 File).

**Fig 3:**
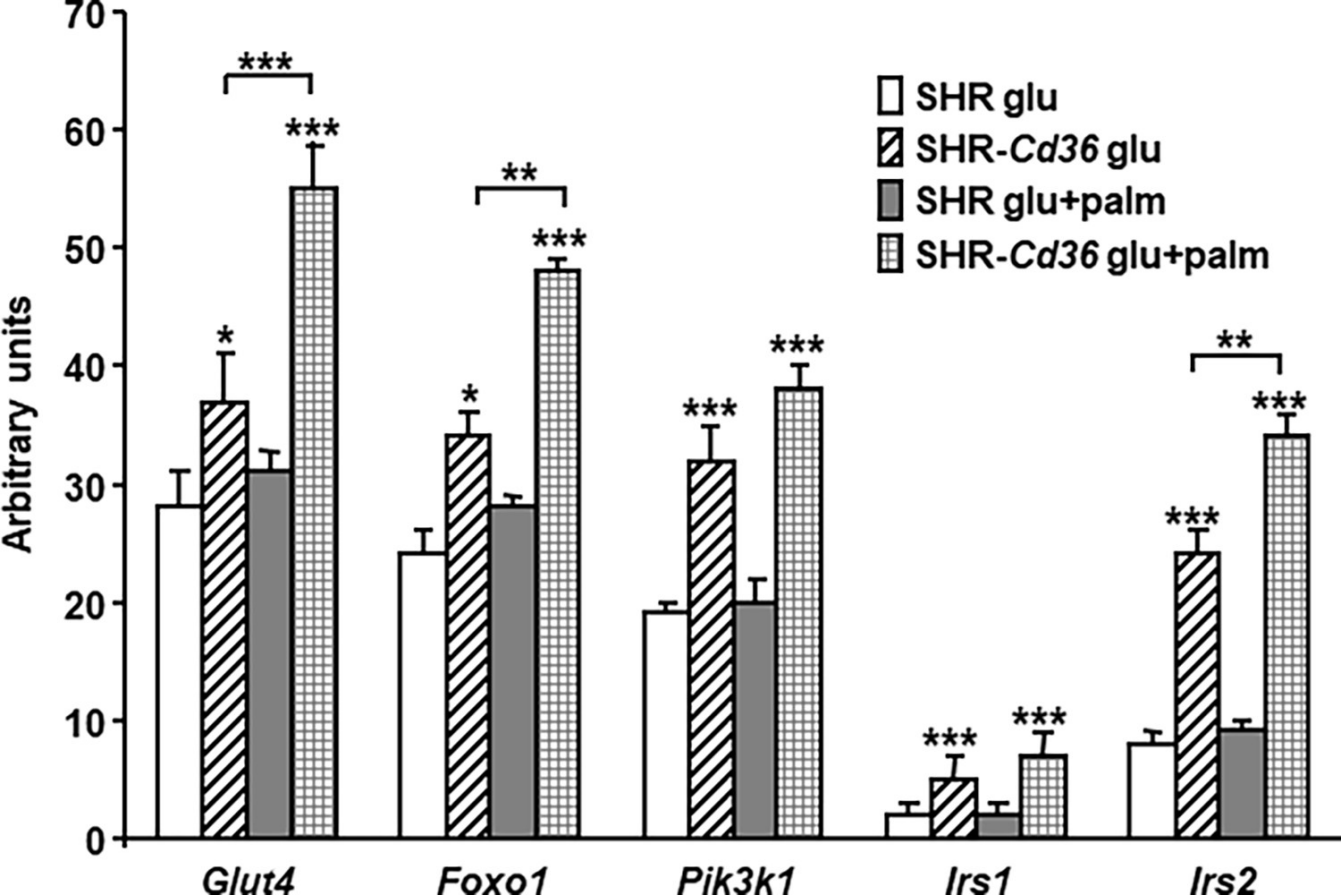
Effect of *Cd36* on expression of genes involved in insulin signalling pathway and glucose metabolism. Two-way ANOVA results: P values of statistical significance for effects strain (*Cd36* genotype), type of incubation media (glucose/palmitate) and strain x substrate interaction. For pairwise multiple comparison procedures Holm Sidak testing was used. *, **, and *** denote P<0.05, P<0.01, and P<0.001 significant differences.

As can be seen in Fig 3, the SHR showed similar mRNA expression in of *Irs1* (Insulin receptor substrate 1), *Irs2* (Insulin receptor substrate 2), *Pik3r1* (Phosphoinositide-3-kinase regulatory subunit 1), *Foxo1* (Forkhead box O1) and *Slc4a2* (Solute carrier family 4 member 2, also known as *Glut4*) genes in BAT incubated in media with either glucose alone or glucose + palmitate. On the other hand, SHR-*Cd36* transgenic rats versus SHR had significantly increased expression of these genes when BAT was incubated in media with glucose alone and this difference was even higher when palmitate was added to incubation media (Fig 3).

### Effects of *Cd36* on blood capillary number in BAT and weight of BAT

**Fig 4:**
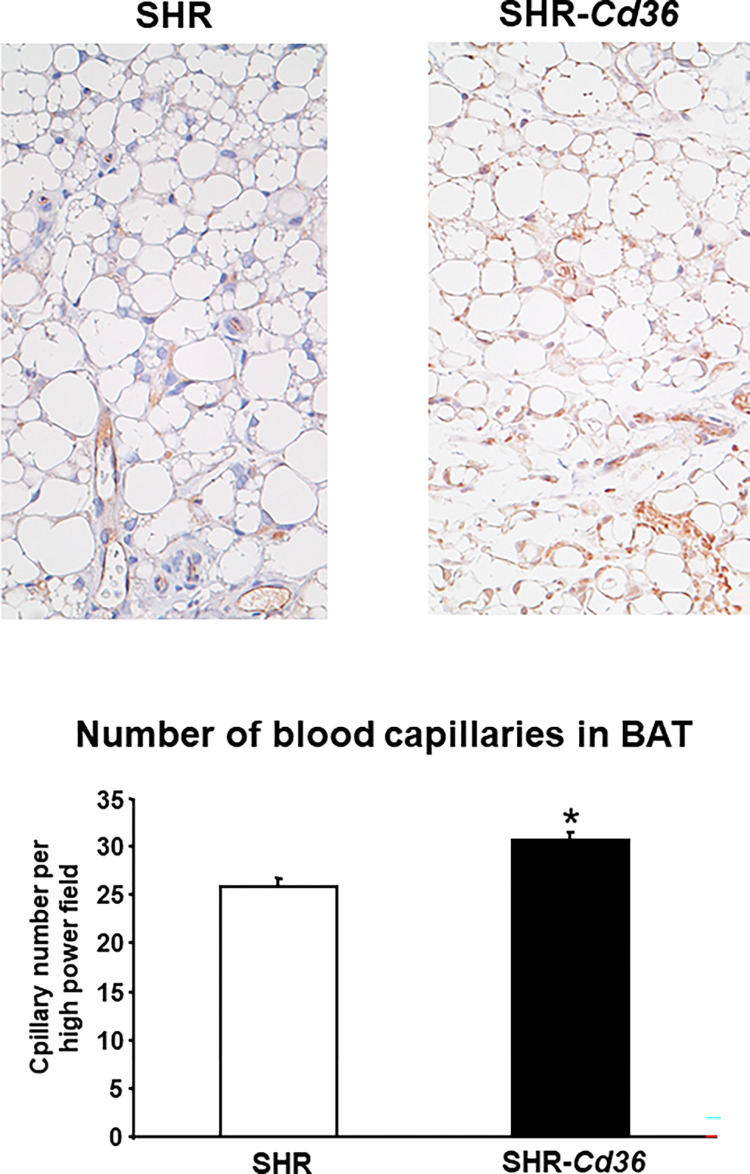
Capillary number in BAT was significantly increased in SHR-*Cd36* versus SHR rats. * denotes P<0.003.

SHR-*Cd36* transgenic rats when compared to the SHR exhibited significantly increased blood capillary number in BAT (Fig 4). In addition, SHR-*Cd36* transgenic rats showed lower relative BAT weight but the difference was not statistically significant (0.109±0.005 vs. 0.096±0.003 g/100 g body weight, P = 0.055).

## Discussion

In the current study, we analysed the role of CD36 fatty acid translocase in regulation of glucose and fatty acid utilisation in BAT using in vitro assay. It should be noted that in vitro assay has a limitation since it does not reflect whole body metabolism. However, in vitro method has also some advantages. Labeled glucose is used for de novo lipogenesis (DNL) not only in BAT but also in other tissues (WAT and liver) in vivo. Labeled fatty acids produced by DNL in WAT and liver will enter BAT. Thus labeled fatty acids in BAT will reflect both DNL and incorporation of fatty acids produced in other tissues. On the other hand, in vitro incubation of BAT from SHR versus SHR-*Cd36* with labeled glucose will enable testing the role of *Cd36* in DNL without confounding factors such as incorporation of labeled fatty acids produced by DNL in other tissues and differences in other systemic parameters (e.g. circulating fatty acids, triglycerides or glucose) between SHR and SHR-*Cd36* that could affect DNL. Our study was focused on the specific role of *Cd36* in glucose and palmitate transport and utilisation in BAT. We did not plan to study the role of *Cd36* in BAT on whole body metabolism.

Our results showed that wild type versus mutant *Cd36* on the SHR genetic background enhanced glucose transport and utilisation and direct oxidation of exogenous palmitate in BAT. However, several studies reported opposite effects of *Cd36* on glucose transport into tissues. For instance, it was found that *Cd36* knockout versus wild type mice showed reduced uptake and oxidation of fatty acids in skeletal muscle while glucose transport into skeletal muscle and glucose oxidation rates were significantly increased [16, 17]. In addition, mice with deletion of *Cd36* specifically in endothelial cells showed reduced fatty acid uptake but increased glucose transport into BAT [18]. It is possible that these discrepant results on the impact of *Cd36* deficiency on glucose transport into tissues may be dependent on nutrient state, tissue specificity and interspecies differences.

A recent study by Samovski et al. [19] showed that CD36 regulates insulin signalling by promoting tyrosine phosphorylation of IRβ by Fyn kinase in the muscle, suggesting that the modulation of IRβ phosphorylation is a key mechanism for CD36-mediated insulin signal transduction. In addition, Yang et al. [20] reported that CD36 deficiency is associated withled to abnormally increased hepatic protein-tyrosine phosphatase 1B (PTP1B) expression in the liver and thatenhanced interaction of PTP1B withand IR interactions might, which contributed to reduceddecreased insulin signalling. Contrary to these findings, we did not observe significant changes in the expression and phosphorylation of IRβ protein but significantly reduced expression and phosphorylation of GSK-3β protein in SHR-*Cd36* transgenic rats. GSK-3 has been implicated as a negative regulator of insulin signalling through serine phosphorylation of IRS-1. GSK-3 is constitutively active and downregulated by phosphorylation mediated by AKT [21]. In addition, it has been reported that GSK-3 phosphorylation is regulated by CD36 when insulin-stimulated phosphorylation of GSK-3 was significantly higher in myotubes with CD36 knockdown [22]. Accordingly, it is possible that CD36 modulates insulin signalling via GSK-3. GSK-3 has been also found to also reduce the thermogenic program in brown adipocytes and inhibition of GSK-3 also caused increased *Ucp1* expression and oxygen consumption [23].

It is widely accepted that fatty acids derived by lipogenesis from glucose in brown adipocytes or transported from circulation are not directly used for UCP1 mediated thermogenesis but preferentially stored in intracellular triglycerides from which fatty acids are provided during thermogenesis [13, 24–26]. Contrary to these reports, our results showed that exogenously provided palmitate can be directly oxidised in the presence of wild type *Cd36*. Recently, Shin et al. [27] demonstrated in mice lacking *Abhd5* (abhydrolase domain containing 5) gene (also known as CGI-58), a lipolytic activator that is essential for the stimulated lipid droplet lipolysis, specifically in BAT or WAT or in both adipose tissues, that BAT lipolysis in not essential for thermogenesis. On the other hand, fasted mice lacking *Abhd5* gene in both BAT and WAT were cold sensitive which suggested an essential role of WAT lipolysis in fueling thermogenesis during fasting. These results provided evidence that brown adipocytes may directly use fatty acids derived from the blood as thermogenic substrates and are congruent with our finding about direct oxidation of exogenous palmitate in BAT in the presence of wild type *Cd36*.

BAT is one of the most vascularised tissues in the body and vasculature has multiple functions in the modulation of BAT functions [28]. For instance, higher metabolic activity in BAT requires increased blood perfusion to supply oxygen and substrates and to export heat, which could be provided by increased blood flow. Our results showed that expression of wild type *Cd36* in BAT was associated with increased blood capillary number despite the fact that CD36 is considered to be a negative regulator of angiogenesis [29].

It can be concluded that *Cd36* in BAT plays an important role (1) in glucose transport and utilisation for lipogenesis via reducing expression and phosphorylation of GSK-3β and (2) in transport and direct oxidation of exogenous fatty acids.

## Supporting information

S1 File Original uncropped images of western blots and Ponceau S staining used for determination of IRβ, phospho-IRβ, PI3K, Akt, phospho-Akt, GSK-3β, and phospho-GSK-3β. (PDF)

S1 Table Primers for testing expression of selected genes involved in insulin signalling and glucose metabolism. (PDF)
