# Supplementary material for: CD36 regulates substrates utilisation in brown adipose tissue of spontaneously hypertensive rats: In vitro study
Source: PLoS One. 2023 Apr 13;18(4):e0283276. doi: 10.1371/journal.pone.0283276 (PMC10101526; doi:10.1371/journal.pone.0283276)
Supplement: S1 File — (PDF) [file pone.0283276.s001.pdf]

S1 File. Original uncropped images of western blots and Ponceau S staining used for determination of IRβ, phospho-IRβ, PI3K, Akt, phospho-Akt, GSK-3β, and phospho-GSK-3β.

S1 Fig. Western blot: (A) membrane značená with IRβ-specific antibody and (B) original uncropped gel stained by Ponceau S.

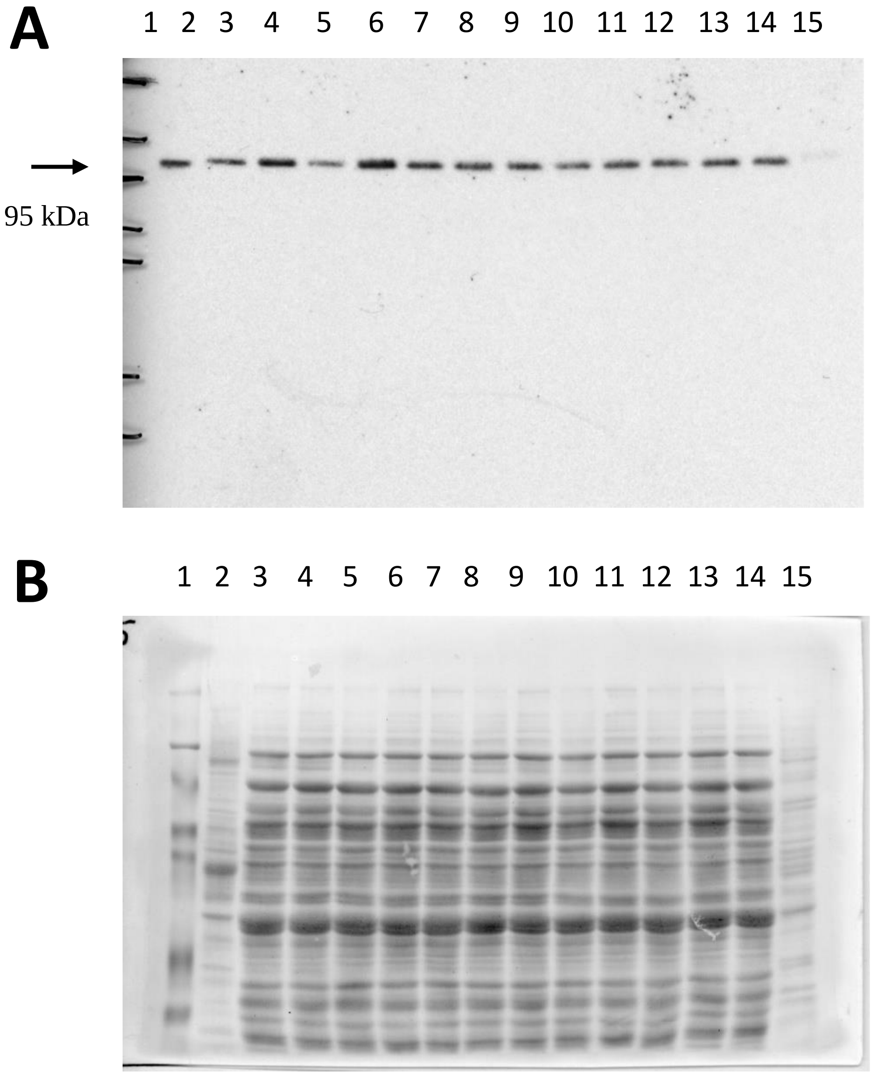

Lane 1: low molecular weight protein markers SDS7B2 from Sigma-Aldrich (26,600 Da, 36,500 Da, 48,500 Da, 58,000 Da, 90,000 Da, 116,000 Da, and 180,000 Da)
Lane 2: cells lysate of H9c2 cells
Lane 3, 7, and 11: BAT lysate from SHR incubated with glucose
Lane 4, 8, and 12: BAT lysate from SHR-*Cd36* incubated with glucose
Lane 5, 9, and 13: BAT lysate from SHR incubated with glucose and palmitate
Lane 6, 10, and 14: BAT lysate from SHR-*Cd36* incubated with glucose and palmitate
Lane 15: cells lysate of HL1 cells

S2 Fig. Western blot (A) and Ponceau S staining (B) for phospho-IRβ.

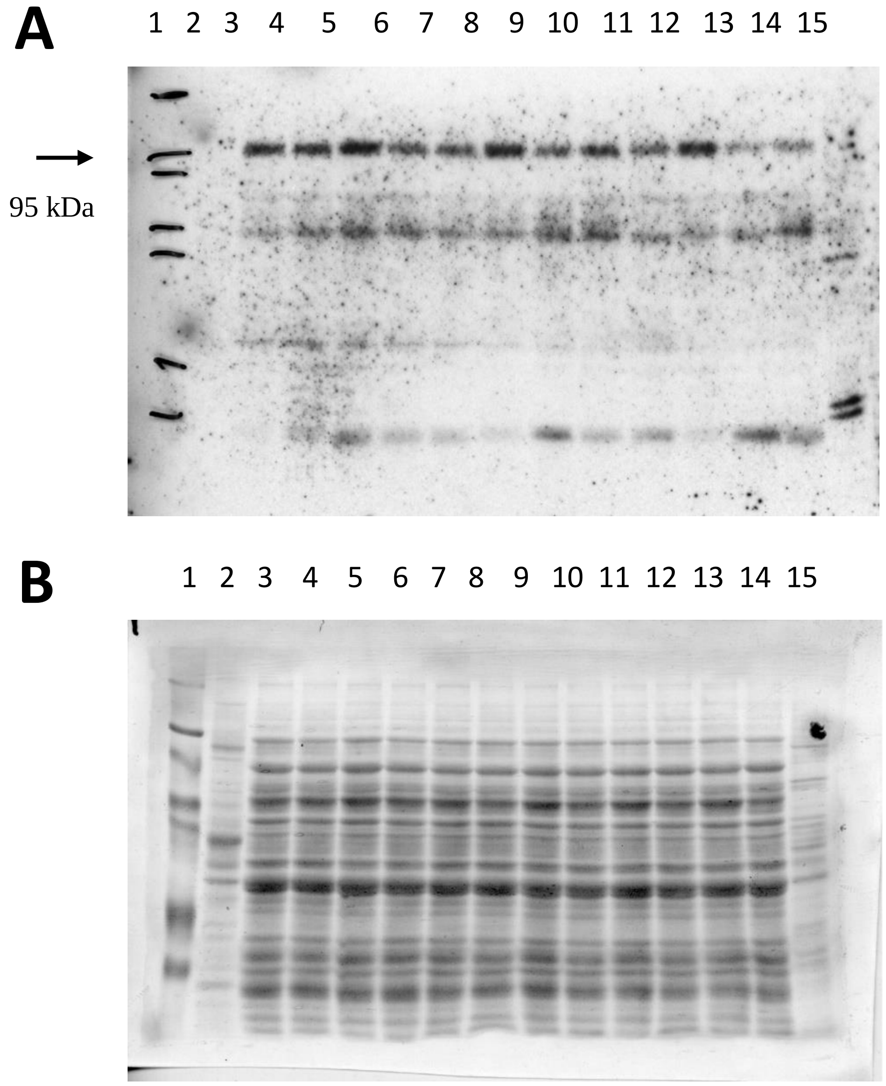

Lane 1: low molecular weight protein markers
Lane 2: cells lysate of H9c2 cells
Lane 3, 7, and 11: BAT lysate from SHR incubated with glucose
Lane 4, 8, and 12: BAT lysate from SHR-*Cd36* incubated with glucose
Lane 5, 9, and 13: BAT lysate from SHR incubated with glucose and palmitate
Lane 6, 10, and 14: BAT lysate from SHR-*Cd36* incubated with glucose and palmitate
Lane 15: cells lysate of HL1 cells

S3 Fig. Western blot (A) and Ponceau S staining (B) for PI3K.

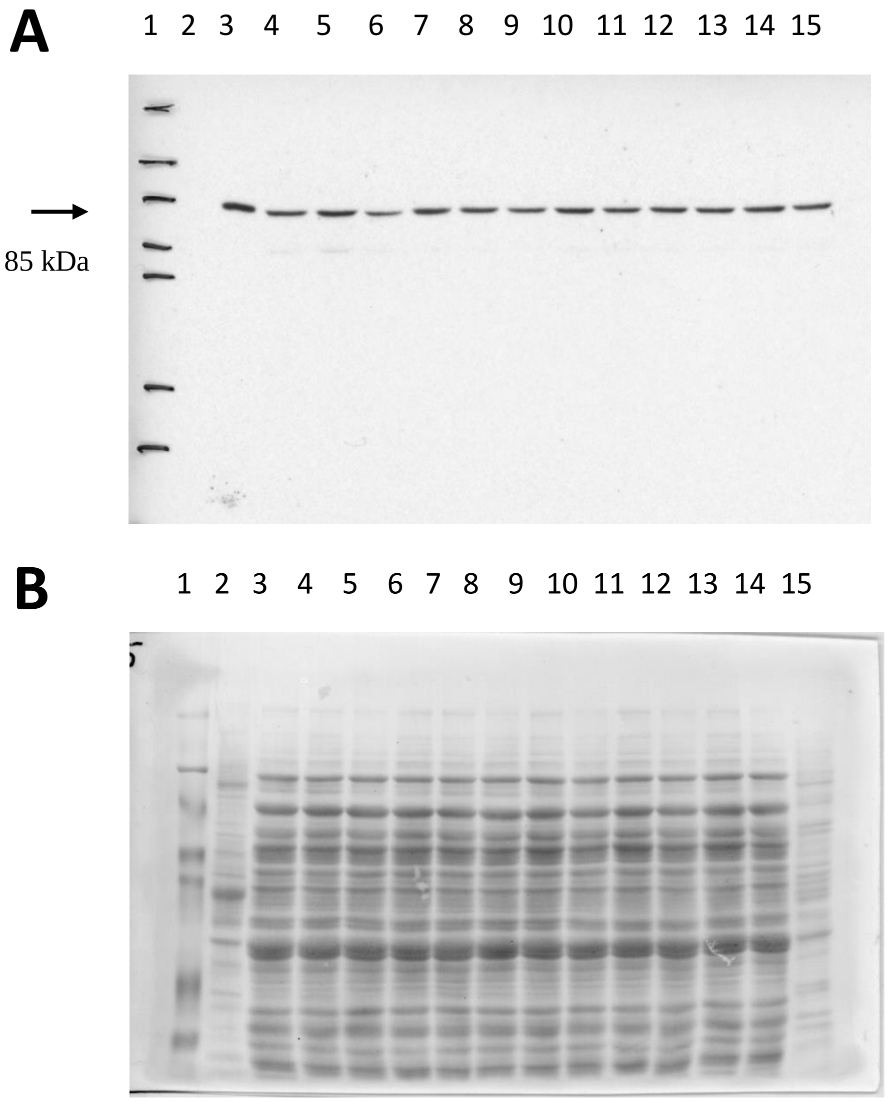

Lane 1: low molecular weight protein markers
Lane 2: cells lysate of H9c2 cells
Lane 3, 7, and 11: BAT lysate from SHR incubated with glucose
Lane 4, 8, and 12: BAT lysate from SHR-*Cd36* incubated with glucose
Lane 5, 9, and 13: BAT lysate from SHR incubated with glucose and palmitate
Lane 6, 10, and 14: BAT lysate from SHR-*Cd36* incubated with glucose and palmitate
Lane 15: cells lysate of HL1 cells

S4 Fig. Western blot (A) and Ponceau S staining (B) for Akt.

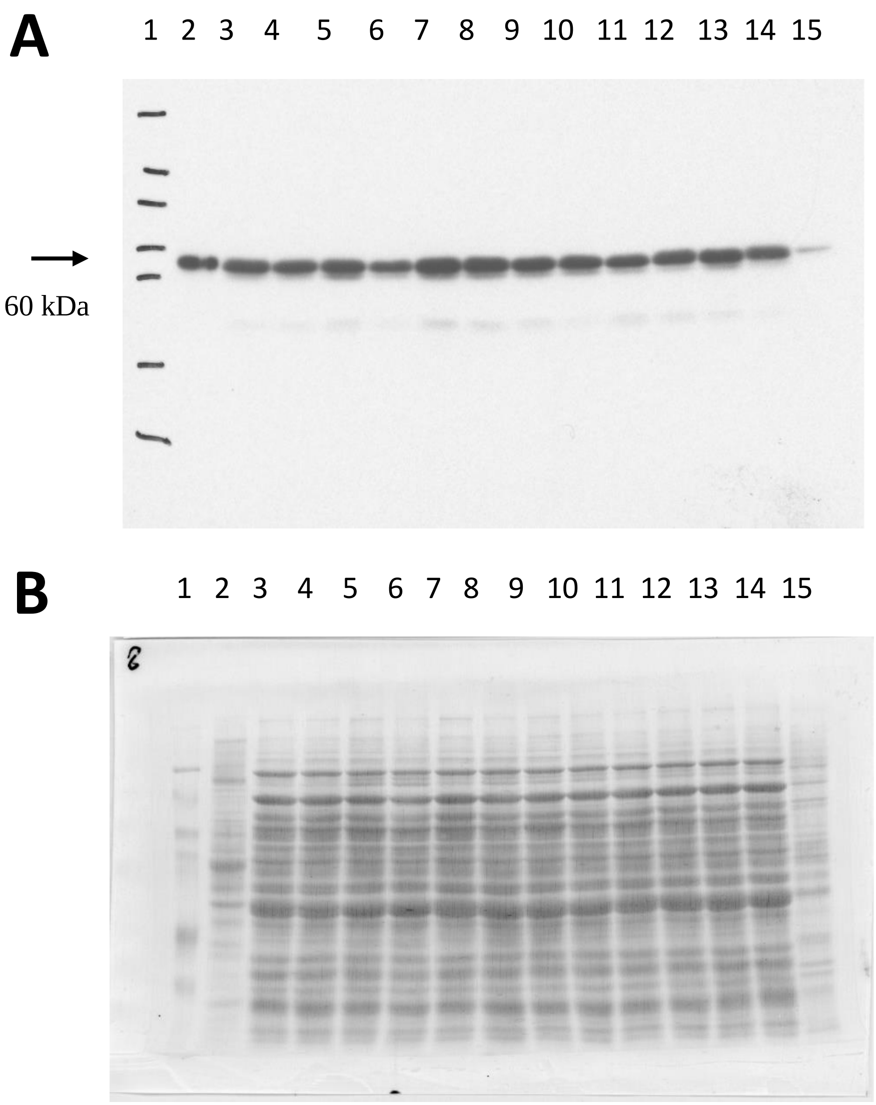

Lane 1: low molecular weight protein markers
Lane 2: cells lysate of H9c2 cells
Lane 3, 7, and 11: BAT lysate from SHR incubated with glucose
Lane 4, 8, and 12: BAT lysate from SHR-*Cd36* incubated with glucose
Lane 5, 9, and 13: BAT lysate from SHR incubated with glucose and palmitate
Lane 6, 10, and 14: BAT lysate from SHR-*Cd36* incubated with glucose and palmitate
Lane 15: cells lysate of HL1 cells

S5 Fig. Western blot (A) and Ponceau S staining (B) for phospho-Akt.

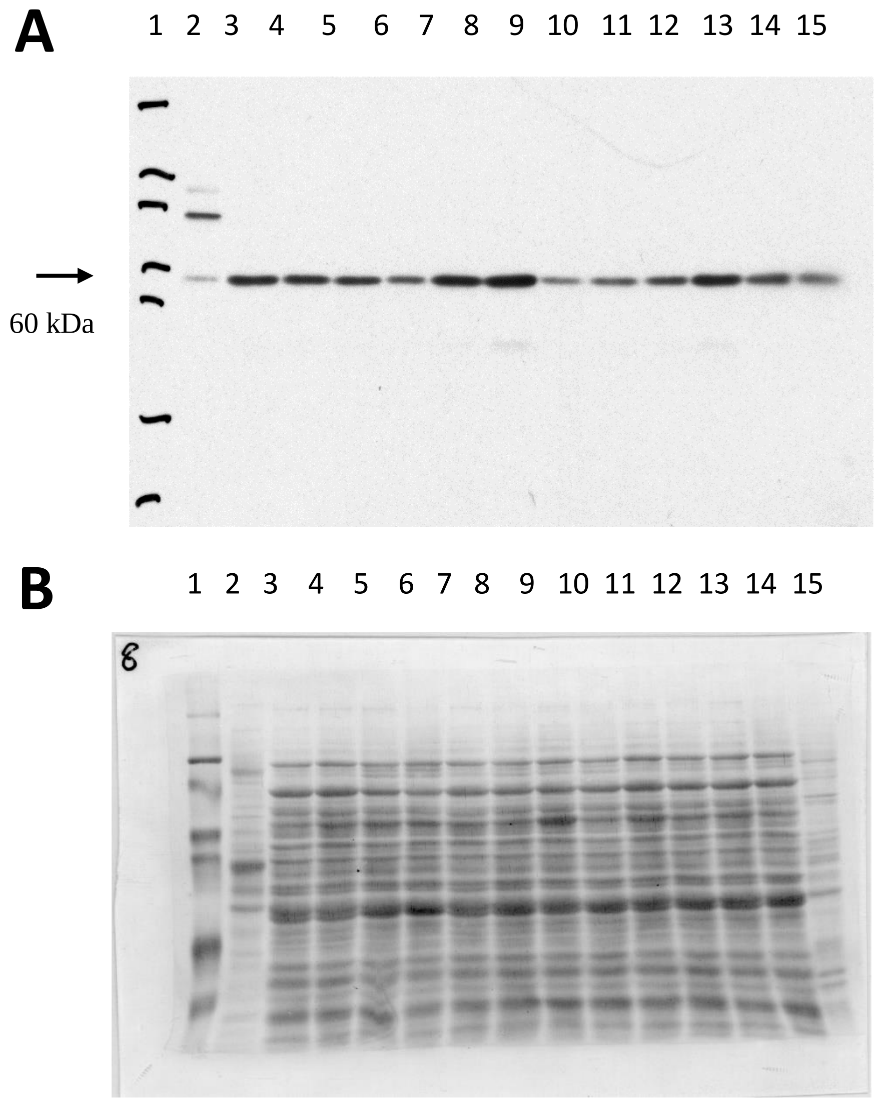

Lane 1: low molecular weight protein markers
Lane 2: cells lysate of H9c2 cells
Lane 3, 7, and 11: BAT lysate from SHR incubated with glucose
Lane 4, 8, and 12: BAT lysate from SHR-*Cd36* incubated with glucose
Lane 5, 9, and 13: BAT lysate from SHR incubated with glucose and palmitate
Lane 6, 10, and 14: BAT lysate from SHR-*Cd36* incubated with glucose and palmitate
Lane 15: cells lysate of HL-1 cells

S6 Fig. Western blot (A) and Ponceau S staining (B) for GSK-3β.

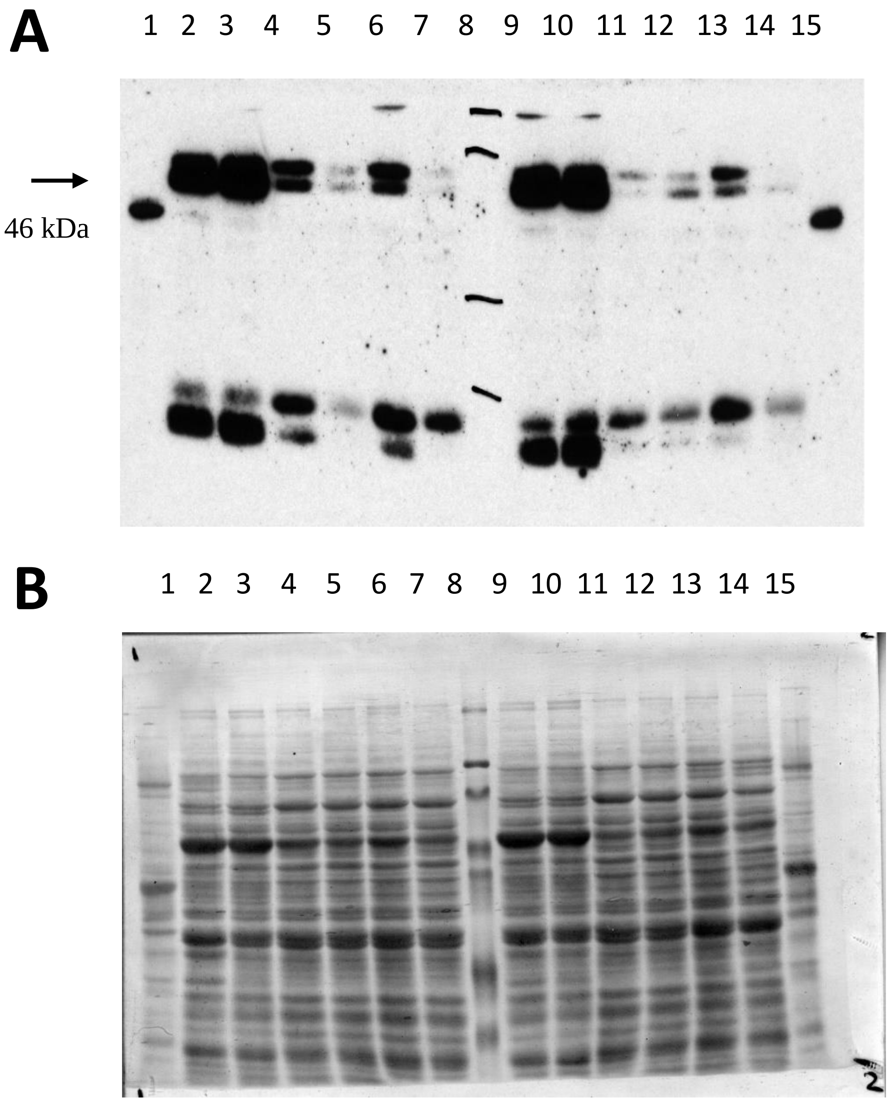

Lane 1 and 15: cells lysate of H9c2 cells
Lane 2 and 9: heart lysate from SHR
Lane 3 and 10: herat lysate from SHR-*Cd36*
Lane 4 and 11: BAT lysate from SHR incubated with glucose
Lane 5 and 12: BAT lysate from SHR-*Cd36* incubated with glucose
Lane 6 and 13: BAT lysate from SHR incubated with glucose and palmitate
Lane 7 and 14: BAT lysate from SHR-*Cd36* incubated with glucose and palmitate
Lane 8: low molecular weight protein marker

S7 Fig. Western blot (A) and Ponceau S staining (B) for phospho-GSK-3β.

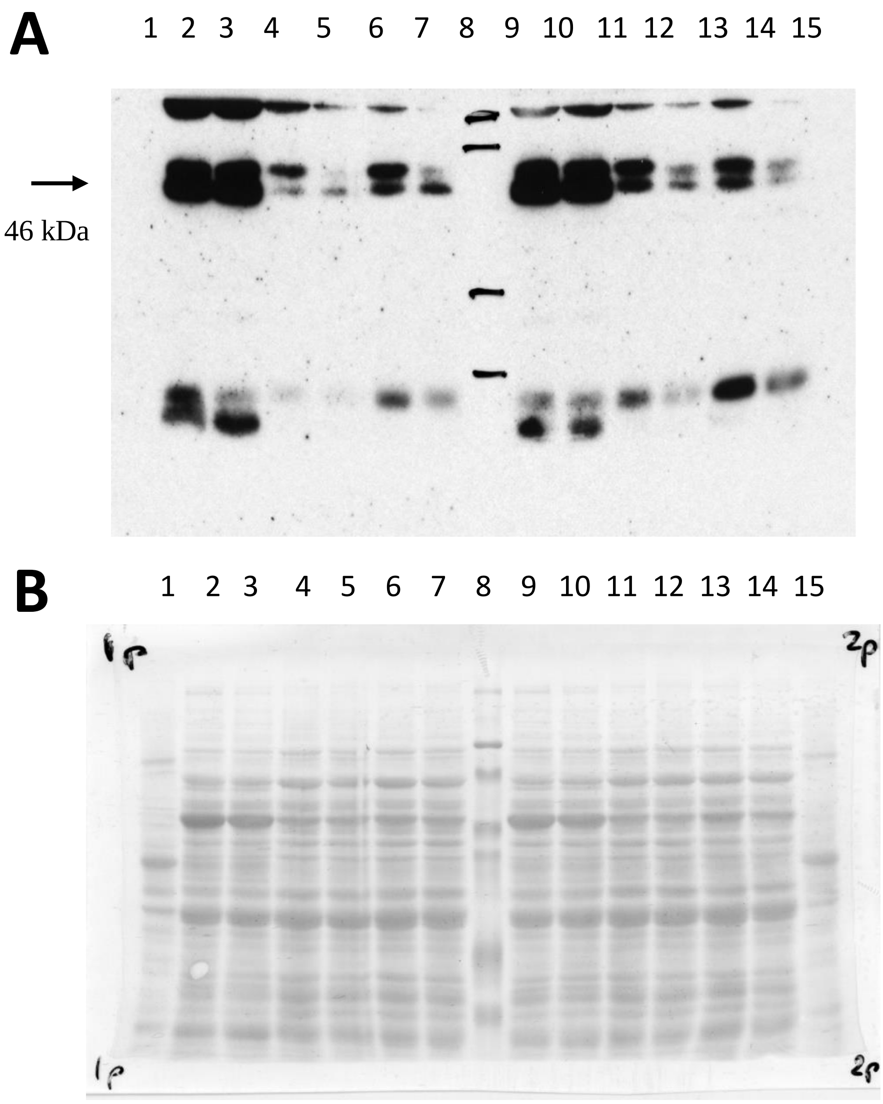

Lane 1 and 15: cells lysate of H9c2 cells
Lane 2 and 9: heart lysate from SHR
Lane 3 and 10: herat lysate from SHR-*Cd36*
Lane 4 and 11: BAT lysate from SHR incubated with glucose
Lane 5 and 12: BAT lysate from SHR-*Cd36* incubated with glucose
Lane 6 and 13: BAT lysate from SHR incubated with glucose and palmitate
Lane 7 and 14: BAT lysate from SHR-*Cd36* incubated with glucose and palmitate
Lane 8: low molecular weight protein marker
