# Supplementary material for: CD36 regulates substrates utilisation in brown adipose tissue of spontaneously hypertensive rats: In vitro study
Source: PLoS One. 2023 Apr 13;18(4):e0283276. doi: 10.1371/journal.pone.0283276 (PMC10101526; doi:10.1371/journal.pone.0283276)
Supplement: S1 Table — (PDF) [file pone.0283276.s002.pdf]

Supplementary Table 1 Primers for testing expression of selected genes involved in insulin signalling and glucose metabolism.

Genes	Forward primers	Reverse primers
Foxo1	GTGAACACCATGCCTCACAC	CACAGTCCAAGCGCTCAATA
Irs1	TGTGCCAAGCAACAAGAAAG	ACGGTTTCAGAGCAGAGGAA
Irs2	CCCAGAAGATAGCGGGTACA	TGAGAAGAAGTCAGGTGGGG
Pik3k1	GATCAGCGAGATCATCGACA	TGTTTTCGTTTCCCAACCAT
Slc4a2 (Glut4)	CATATTTGGCTTTGTGGCC	TCCAGTTGGAGAAACCAGC
Ppia	AGCATACAGGTCCTGGCAT	TCACCTTCCCAAAGACCAC
